# Synthesis of New 2-Arylbenzo[*b*]furan Derivatives via Palladium-Catalyzed Suzuki Cross-Coupling Reactions in Aqueous Media

**DOI:** 10.3390/molecules23102450

**Published:** 2018-09-25

**Authors:** Qianqian Chen, Panli Jiang, Mengping Guo, Jianxin Yang

**Affiliations:** 1 Laboratory of Green Catalysis and Reaction Engineering of Haikou, Hainan Provincial Fine Chemical Engineering Research Center, College of Chemistry and Chemical Engineering, Hainan University, Haikou 570228, China; chenqq9297@sina.com; 2 Institute of Coordination Catalysis, College of Chemistry and Bio-Engineering, Yichun University, Yichun 336000, China; jiangpanli1991@163.com

**Keywords:** heterobiaryl compounds, palladium(II) complex catalyst, Suzuki cross-coupling, aqueous phase

## Abstract

A series of novel benzofuran derivatives containing biaryl moiety were designed and synthesized by the Suzuki cross-coupling reactions. The reactions, performed in the presence of K_2_CO_3_, EtOH/H_2_O and Pd(II) complex as catalyst, gave the corresponding products in good to excellent yields. The methodology allows the facile production of heterobiaryl compounds, a unique architectural motif that is ubiquitous in medicinal chemistry.

## 1. Introduction

**Figure 1:**
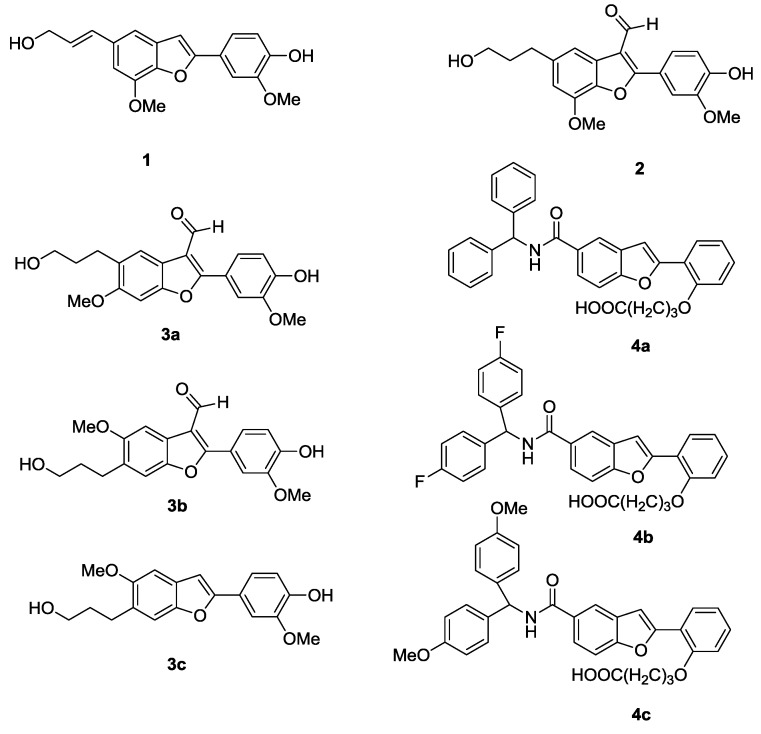
Relevant molecules with a 2-arylbenzo[*b*]furan moiety.

2-Arylbenzo[*b*]furan moiety is a common structural subunit found in natural products [1,2,3] and synthetic compounds with important biological activities [4,5,6,7]. For example, a representative complex of the natural 3-deformylated 2-arylbenzo[*b*]furan is ailanthoidol in (Figure 1, **1**), which was isolated from the chloroform-soluble fraction of the tree of *Zanthoxylum ailanthoides*, was found to have a broad range of biological activities such as anticancer [8], immunosuppressive [9,10,11], antivirus [12,13,14,15], antioxidant [10,11], antifungal [16], and antifeedant activities [17]. Meanwhile, 5-(3-hydroxypropyl-7-methoxy-2-(3′-methoxy-4′-hydroxyphenyl)benzo[*b*]furan-3-carbaldehyde (XH-14) (Figure 1, **2**), which has been widely used in China for the treatment of coronary heart diseases such as myocardial infarction and angina pectoris [18], was isolated from the plant *Salvia miltorrhiza* Bunge (Chinese name “Danshen”). Jun [19] obtained three XH-14 analogues whose anti-inflammatory effects were examined in lipopolysaccharide(LPS)-stimulated RAW 264-7 macrophages. The results showed that three structurally modified derivatives (Figure 1, **3a**–**3c**) inhibited significantly the production of inflammatory mediator nitric oxide without showing cytotoxicity. Moreover, Nishi and coworkers synthesized a series of 2-phenylbenzofuran derivatives with both carboxy and 5- or 6-diphenylmethylcarbamoyl groups (Figure 1, **4a**–**4c**), which showed inhibitory activities against both enzymes and were more active against human type **I** enzyme than against type **II** enzyme [20]. 

Motivated by the above-mentioned 2-arylbenzo[*b*]furan derivatives as valuable building blocks with a wide range of biological activities, to discover new potentially active agents, in this research, a series of novel benzofuran derivatives containing biaryl moiety were designed and synthesized. Biaryls are recurring functional groups in many natural products, pharmaceuticals and bioactive compounds [21,22,23]. Palladium-catalyzed cross-coupling of aryl halides with organoboronic acids, known as the Suzuki cross-coupling reaction, is a versatile and highly utilized reaction for the selective formation of carbon-carbon bonds, in particular for the synthesis of biaryls [24,25,26,27,28]. This paper describes the Suzuki reaction applied to the synthesis of novel benzofuran derivatives containing biaryl moiety. 

## 2. Results and Discussion

**Scheme 1:**
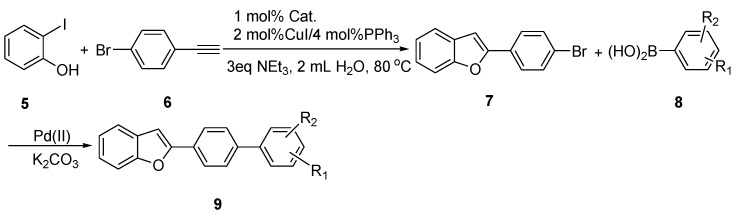
Synthesis of benzofuran derivatives containing biaryl moiety **9**.

The designed novel benzofuran derivatives containing biaryl moiety (**9**) were prepared in two steps (Scheme 1). First, 2-(4-bromophenyl)benzofuran (**7**) was obtained following the method, Pd(II)/CuI/PPh_3_-co-catalyzed coupling-cyclization reaction of the commercially available 2-iodophenol (**5**) with 4-bromo-1-ethynylbenzene (**6**) in the presence of NEt_3_ in water at 80 °C, reported by the Guo group [29]. Second, the optimal reaction conditions were studied by employing the Suzuki cross-coupling of 2-(4-bromophenyl)benzofuran (**7**) with 4-methoxyphenylboronic acid as model reaction for the synthesis of the 2-arylbenzo[*b*]furan derivatives. As can be seen in Table 1, we first examined the catalytic activity using common palladium salts PdCl_2_ or Pd(OAc)_2_ as catalyst in the presence of K_2_CO_3_ in EtOH/H_2_O (1:1) at 80 °C, only moderate yields of 55% or 61% were achieved (Table 1, entries 1–2), but the reaction proceeded well in 91% yield in the presence of our newly developed Pd(II) complex catalyst (**10**) [30] (Table 1, entry 3). Compared to loading of catalyst 1 mol%–4 mol%, the yield was obviously enhanced to 97% when 3 mol% Pd(II) complex catalyst was used (Table 1, entry 5). The effects of base on the reaction were next examined. 28%, 40%, 53%, 78% and 63% yield of the desired product was obtained when using NEt_3_, NaF, NaHCO_3_, NaOH and Cs_2_CO_3_ as a base, respectively (Table 1, entries 7–11). Replacing co-solvent EtOH/H_2_O (1:1) with H_2_O, EtOH, DMF or DMSO further optimized the reaction condition respectively, giving the product in only trace amounts (Table 1, entries 12–15). Further optimizations showed that increasing the reaction time did not improve the reaction outcome (Table 1, entries 17–21) and decreasing reaction temperature obtained poor yields (Table 1, entries 16–17).

Then, under the best conditions, the use of different arylboronic acid for efficient synthesis of new 2-arylbenzo[*b*]furan derivatives was examined. The desired products were obtained in good to excellent yields (92%–98%) with substrates that contained electron-withdrawing and donating groups (Table 2, entries 1–4). The effect of steric hindrance was also tested with *ortho*-substituted boronic acid showing slightly lower yield (85%) (Table 2, entry 5). 

## 3. Experimental

### 3.1. General Information

Commercial reagents employed in the synthesis were analytical grade, obtained from Alfa Aesar (Ward Hill, MA, USA) and used as received without any prior purification. Silica gel GF254 (Qingdao Haiyang Chemical Co., Ltd., Qingdao, China) was used for analytical thin-layer chromatography (TLC) (glass coating 0.25 mm thick) using hexane and dichloromethane as the eluent. ^1^H-NMR, ^13^C-NMR spectra were recorded on a BRUKER DRX (400 MHz) spectrometer (Billerica, MA, USA) using tetramethylsilane as the internal standard and CDCl_3_ or CD_2_Cl_2_ as the solvent. Low-resolution mass-spectra were recorded on an Agilent gas chromatography mass spectrometry 7890A-5795C instrument. High-resolution mass spectra (HRMS) were obtained using Agilent 6210 ESI/TOF mass spectrometer (Santa Clara, CA, USA). Melting points were determined using a Mettler FP5 melting point apparatus (Columbus, OH, USA) in open capillaries and were uncorrected. The ^1^H-NMR, ^13^C-NMR and HRMS for all the synthesized compounds are available in the supplementary materials.

### 3.2. General Procedure for Suzuki Coupling

2-(4-Bromophenyl)benzofuran (0.05 mmol, 0.0137 g), palladium(II) (**10**) (0.0015 mmol, 0.0012 g), K_2_CO_3_ (0.1 mmol, 0.0138 g) and relevant arylboronic acid (0.08 mmol) were dissolved in EtOH + H_2_O (*v*/*v* = 1:1, 6 mL) and the resulting suspension stirred at 80 °C for 4 h. After cooling to ambient temperature brine (10 mL) was added to the mixture, the aqueous layer was extracted with dichloromethane (3 × 10 mL). The combined organic layers were dried (Na_2_SO_4_) and concentrated, and the residue was purified by thin layer chromatography to give the 2-arylbenzo[*b*]furan derivatives **9a**–**9g**.

*2-(4′-Methoxybiphenyl-4-yl)benzofuran* (**9a**). White powder m.p. 270–271 °C; ^1^H-NMR (400 MHz, CDCl_3_): *δ* 7.92 (d, *J* = 8.0 Hz, 2H), 7.65 (d, *J* = 8.0 Hz, 2H), 7.60–7.51 (m, 4H), 7.27–7.25 (m, 2H), 7.04 (s, 1H), 7.01 (d, *J* = 8.0 Hz, 2H), 3.86 (s, 3H). ^13^C-NMR (100 MHz, CDCl_3_): *δ* 159.4, 155.8, 154.9, 140.8, 132.9, 129.3, 128.7, 128.0, 126.9, 125.3, 124.2, 122.9, 120.8, 114.3, 111.1, 101.1, 55.3. GC-MS (EI): 300.1 ([M]^+^). HRMS (ESI) *m*/*z:* calcd for C_21_H_16_O_2_ [M + H]^+^ 301.1223; found 301.1227.

*1-(4′-Benzofuran-2-ylbiphenyl-4-yl)ethanone* (**9b**). White powder m.p. 273–275 °C; ^1^H-NMR (400 MHz, CD_2_Cl_2_): *δ* 7.97 (d, *J* = 8.0 Hz, 2H), 7.91 (d, *J* = 8.0 Hz, 2H), 7.69 (d, *J* = 8.0 Hz, 4H), 7.55 (d, *J* = 8.0 Hz, 1H), 7.47 (d, *J* = 8.0 Hz, 1H), 7.25–7.15 (m, 2H), 7.05 (s, 1H), 2.53 (s, 3H). ^13^C-NMR (100 MHz, CD_2_Cl_2_): *δ* 197.2, 155.3, 155.0, 144.6, 139.7, 136.1, 130.2, 129.1, 128.9, 127.5, 126.9, 125.3, 124.5, 123.0, 120.9, 111.0, 101.9, 26.4. GC-MS (EI): 312.2 ([M]^+^). HRMS (ESI) *m*/*z:* calcd for C_22_H_16_O_2_ [M + H]^+^ 313.1223; found 313.1219.

*2-(4′-Propylbiphenyl-4-yl)benzofuran* (**9c**). Pale yellow solid m.p. 244–246 °C; ^1^H NMR (400 MHz, CDCl_3_): *δ* 7.91 (d, *J* = 8.4 Hz, 2H), 7.66 (d, *J* = 8.5 Hz, 2H), 7.57 (d, *J* = 10.0 Hz, 2H), 7.53 (dd, *J* = 9.3, 1.3 Hz, 2H), 7.32–7.19 (m, 4H), 7.03 (d, *J* = 0.5 Hz, 1H), 2.70–2.56 (m, 2H), 1.77–1.59 (m, 2H), 0.98 (t, *J* = 7.3 Hz, 3H). ^13^C NMR (100 MHz, CDCl_3_): *δ* 155.80, 154.93, 142.25, 141.21, 137.75, 129.30, 129.08, 128.99, 127.23, 126.78, 125.31, 124.22, 122.94, 120.85, 111.15, 101.26, 37.72, 24.54, 13.88. GC-MS (EI): 312.1 ([M]^+^). HRMS (ESI) *m*/*z:* calcd for C_23_H_20_O [M + H]^+^ 313.1587; found 313.1593.

*2-(3′-Methylbiphenyl-4-yl)benzofuran* (**9d**). Pale yellow solid mp 168–169 °C (lit. 162–164 °C [7]); ^1^H-NMR (400 MHz, CDCl_3_): *δ* 7.93 (d, *J* = 8.0 Hz, 2H), 7.68 (d, *J* = 8.0 Hz, 2H), 7.59 (d, *J* = 8.0 Hz, 1H), 7.54 (d, *J* = 8.0 Hz, 1H), 7.45 (d, *J* = 8.0 Hz, 2H), 7.35 (t, *J* = 12.0 Hz, 1H), 7.30–7.17 (m, 3H), 7.05 (s, 1H), 2.43 (s, 3H). ^13^C-NMR (100 MHz, CDCl_3_): *δ* 155.7, 154.9, 141.4, 140.4, 138.4, 131.9, 129.3, 128.7, 128.3, 127.7, 127.4, 125.3, 124.2, 124.1, 122.9, 120.8, 111.1, 101.3, 21.5. GC-MS (EI): 284.1 ([M]^+^).

*2-(2′-Methylbiphenyl-4-yl)benzofuran* (**9e**). Pale yellow solid m.p. 80–81 °C; ^1^H-NMR (400 MHz, CDCl_3_): *δ* 7.84 (d, *J* = 8.0 Hz, 2H), 7.53 (d, *J* = 8.0 Hz, 1H), 7.47 (d, *J* = 8.0 Hz, 1H), 7.35 (d, *J* = 8.0 Hz, 2H), 7.23–7.14 (m, 6H), 6.97 (s, 1H), 2.24 (s, 3H). ^13^C-NMR (100 MHz, CDCl_3_): *δ* 154.8, 153.9, 141.2, 140.2, 134.3, 130.9, 129.4, 128.6, 128.2, 127.9, 126.4, 124.8, 123.6, 123.2, 121.9, 119.8, 110.1, 100.2, 19.4. GC-MS (EI): 284.1 ([M]^+^). HRMS (ESI) *m*/*z:* calcd for C_21_H_16_O [M + H]^+^ 285.1274; found 285.1279.

*2-(3′,4′-Difluorobiphenyl-4-yl)benzofuran* (**9f**). White powder m.p. 195–197 °C; ^1^H-NMR (400 MHz, CDCl_3_): *δ* 7.93 (d, *J* = 8.5 Hz, 2H), 7.61 (d, *J* = 8.4 Hz, 2H), 7.60 (d, *J* = 8.3 Hz, 1H), 7.54 (d, *J* = 8.0 Hz, 1H), 7.43 (ddd, *J* = 11.5, 7.5, 2.2 Hz, 1H), 7.38–7.33 (m, 1H), 7.33–7.28 (m, 1H), 7.28–7.19 (m, 2H), 7.07 (s, 1H). ^13^C-NMR (100 MHz, CDCl_3_): *δ* 155.27 (s), 154.96 (s), 150.58 (dd, *J* = 248.0, 12.8 Hz), 150.07 (dd, *J* = 248.8, 12.8 Hz), 139.01 (s), 137.54 (dd, *J* = 5.9, 3.9 Hz), 129.94 (s), 129.15 (s), 127.27 (s), 125.44 (s), 124.49 (s), 123.05 (s), 122.87 (dd, *J* = 6.2, 3.5 Hz), 120.97 (s), 117.64 (d, *J* = 17.2 Hz), 115.84 (d, *J* = 17.7 Hz), 111.20 (s), 101.78 (s). GC-MS (EI): 306.1 ([M]^+^). HRMS (ESI) *m*/*z:* calcd for C_20_H_12_F_2_O [M + Na]^+^ 329.0748; found 329.0751.

*2-(3′,5′-Difluorobiphenyl-4-yl)benzofuran* (**9g**). White powder m.p. 163–165 °C; ^1^H-NMR (400 MHz, CDCl_3_): *δ* 7.93 (d, *J* = 8.5 Hz, 2H), 7.62 (d, *J* = 8.6 Hz, 2H), 7.59 (dd, *J* = 8.0, 0.7 Hz, 1H), 7.53 (br d, *J* = 8.0 Hz, 1H), 7.35–7.27 (m, 1H), 7.27–7.21 (m, 1H), 7.19–7.09 (m, 2H), 7.07 (s, 1H), 6.80 (tt, *J* = 8.8, 2.3 Hz, 1H). ^13^C-NMR (100 MHz, CDCl_3_): *δ* 163.38 (dd, *J* = 248.2, 13.1 Hz), 155.15 (s), 155.01 (s), 143.72 (t, *J* = 9.5 Hz), 138.69 (t, *J* = 2.5 Hz), 130.55 (s), 129.13 (s), 127.33 (s), 125.45 (s), 124.58 (s), 123.08 (s), 121.02 (s), 111.23 (s), 109.96–109.52 (m), 102.75 (t, *J* = 25.4 Hz), 102.02 (s). GC-MS (EI): 306.1 ([M]^+^). HRMS (ESI) *m*/*z:* calcd for C_20_H_12_F_2_O [M + H]^+^ 307.0929; found 307.0934.

## 4. Conclusions

In summary, a series of novel benzofuran derivatives containing biaryl moiety were designed and synthesized. This work establishes that 2-(4-bromophenyl)benzofuran are suitable substrates for Suzuki cross-coupling reactions with relevant arylboronic acids. We found that in the presence of Pd(II) (**10**) as palladium catalyst, the Suzuki reactions proceed in relatively good yields in aqueous medium. This could provide a promising access to new heterobiaryl compounds, valuable building blocks for use in medicinal chemistry.

## Figures and Tables

**Table 1:**
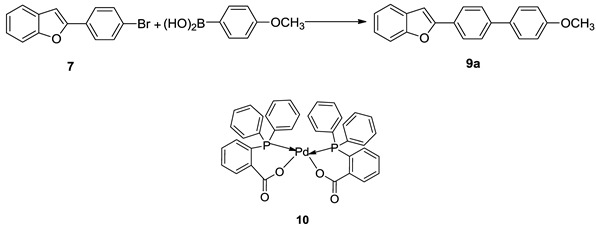
Screening of reaction conditions ^a^.

Entry	Catalyst	Loading of Catalyst (mol%)	Base (mmol)	Solvent (mL) (1:1)	Temperature (°C)	Time (h)	Yield ^b^ (%)
1	PdCl_2_	2	K_2_CO_3_	EtOH + H_2_O	80	4	55
2	Pd(OAc)_2_	2	K_2_CO_3_	EtOH + H_2_O	80	4	61
3	Pd(II) (10)	2	K_2_CO_3_	EtOH + H_2_O	80	4	91
4	Pd(II) (10)	1	K_2_CO_3_	EtOH + H_2_O	80	4	62
5	Pd(II) (10)	3	K_2_CO_3_	EtOH + H_2_O	80	4	97
6	Pd(II) (10)	4	K_2_CO_3_	EtOH + H_2_O	80	4	95
7	Pd(II) (10)	3	NEt_3_	EtOH + H_2_O	80	4	28
8	Pd(II) (10)	3	NaF	EtOH + H_2_O	80	4	40
9	Pd(II) (10)	3	KHCO_3_	EtOH + H_2_O	80	4	53
10	Pd(II) (10)	3	NaOH	EtOH + H_2_O	80	4	78
11	Pd(II) (10)	3	Cs_2_CO_3_	EtOH + H_2_O	80	4	63
12	Pd(II) (10)	3	K_2_CO_3_	EtOH	80	4	32
13	Pd(II) (10)	3	K_2_CO_3_	H_2_O	80	4	0
14	Pd(II) (10)	3	K_2_CO_3_	DMSO	80	4	0
15	Pd(II) (10)	3	K_2_CO_3_	DMF	80	4	trace
16	Pd(II) (10)	3	K_2_CO_3_	EtOH + H_2_O	40	4	13
17	Pd(II) (10)	3	K_2_CO_3_	EtOH + H_2_O	60	4	47
18	Pd(II) (10)	3	K_2_CO_3_	EtOH + H_2_O	80	1	71
19	Pd(II) (10)	3	K_2_CO_3_	EtOH + H_2_O	80	2	93
20	Pd(II) (10)	3	K_2_CO_3_	EtOH + H_2_O	80	3	95
21	Pd(II) (10)	3	K_2_CO_3_	EtOH + H_2_O	80	5	98

^a^ Reaction conditions: 0.05 mmol 2-(4-bromophenyl)benzofuran, 0.08 mmol 4-methoxyphenylboronic acid, 0.1 mmol base, 6 mL solvent, in air. ^b^ Isolated yield.

**Table 2:** Synthesis of new 2-arylbenzo[*b*]furan derivatives ^a^.

Entry	Arylboronic Acid (8)	Product (9)	Yield ^b^ (%)
1	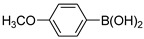 **8a**	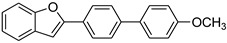 **9a**	97
2	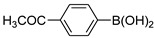 **8b**	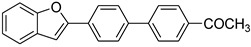 **9b**	97
3	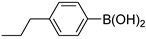 **8c**	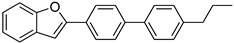 **9c**	92
4	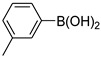 **8d**	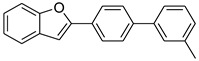 **9d**	95
5	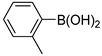 **8e**	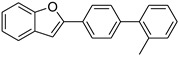 **9e**	85
6	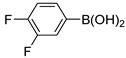 **8f**	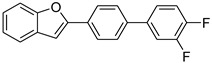 **9f**	78
7	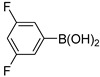 **8g**	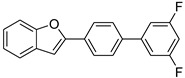 **9g**	96

^a^ Reaction conditions: 0.05 mmol 2-(4-bromophenyl)benzofuran, 0.08 mmol arylboronic acid, 0.1 mmol K_2_CO_3_, 3% mmol Pd(II) (**10**), 6 mL EtOH + H_2_O (1:1), 80 °C, 4 h, in air. ^b^ Isolated yield.

## Supplementary Materials

The ^1^H-NMR, ^13^C-NMR and HRMS for all the synthesized compounds are available online.
