# Supplementary material for: Synthesis of New 2-Arylbenzo[b]furan Derivatives via Palladium-Catalyzed Suzuki Cross-Coupling Reactions in Aqueous Media
Source: Molecules. 2018 Sep 25;23(10):2450. doi: 10.3390/molecules23102450 (PMC6222736; doi:10.3390/molecules23102450)
Supplement: Supplementary file 1 [file molecules-23-02450-s001.pdf]

Synthesis of New 2-Arylbenzo[b]furan Derivatives via Palladium-Catalyzed Suzuki Cross Coupling Reactions in Aqueous Media

Supporting Information

Qianqian Chen, Panli Jiang, Mengping Guo* and Jianxin Yang*

[1] Hainan Provincial Fine Chemical Engineering Research Center, Laboratory of Green Catalysis and Reaction Engineering of Haikou, College of Chemistry and Chemical Engineering, Hainan University, 570228, China;

[2] Institute of Coordination Catalysis, College of Chemistry and Bio-Engineering, Yichun University, Yichun 336000, China;

Characterization

1. ^{1}H-NMR, ^{13}C-NMR, HRMS and GC-MS of 2-(4'-Methoxy-biphenyl-4-yl)-benzofuran (solvent:chloroform-d)

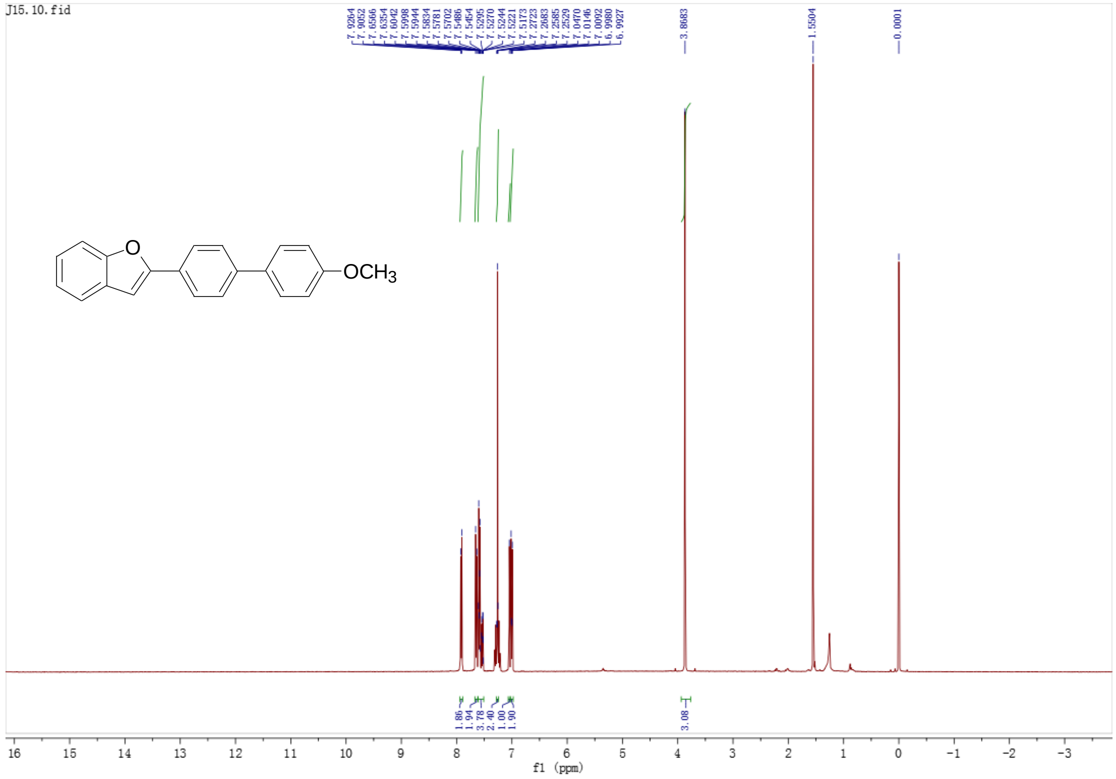

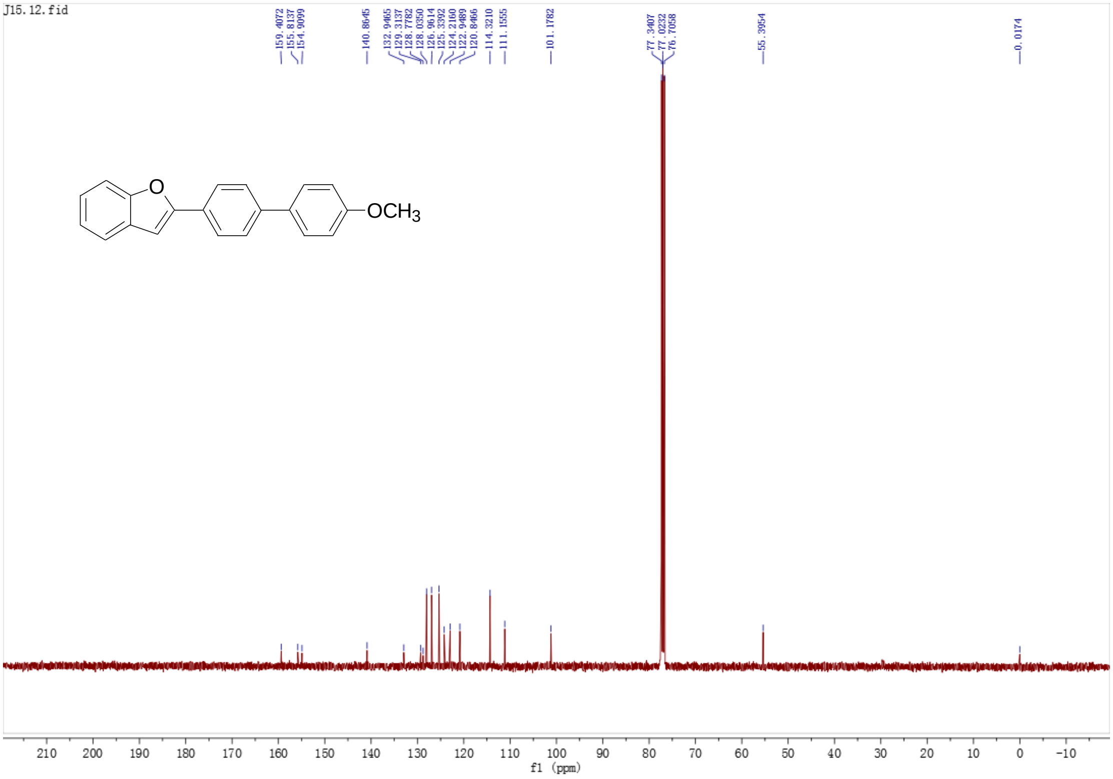
J15.12.fid
159.4072
155.8137
154.9099
140.8645
132.9465
129.3137
128.7782
128.0350
126.9614
125.3392
124.2160
122.9489
120.8466
114.3210
111.1555
101.1782
77.3407
77.0232
76.7058
55.3954
0.0174
OCH3
O
f1 (ppm)

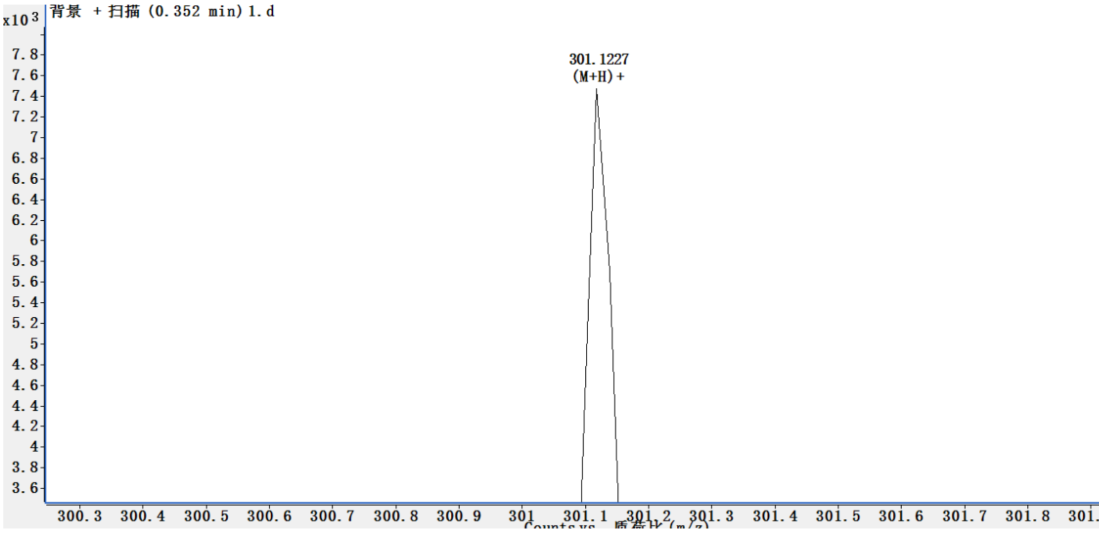
x10 3
背景 + 扫描 (0.352 min) 1.d
301.1227
(M+H)+

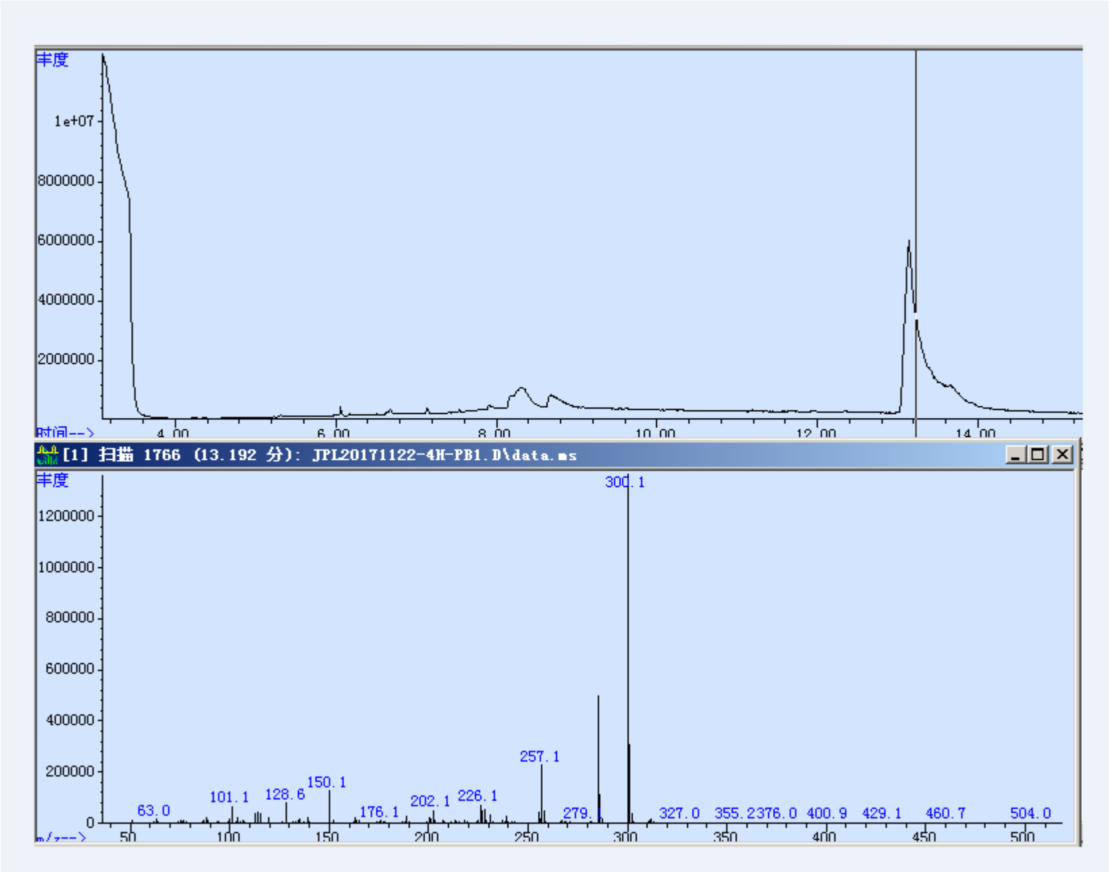

2. ^{1}H-NMR, ^{13}C-NMR, HRMS and GC-MS of 1-(4'-Benzofuran-2-yl-biphenyl-4-yl)-ethanone (solvent: methylene chloride-d2)

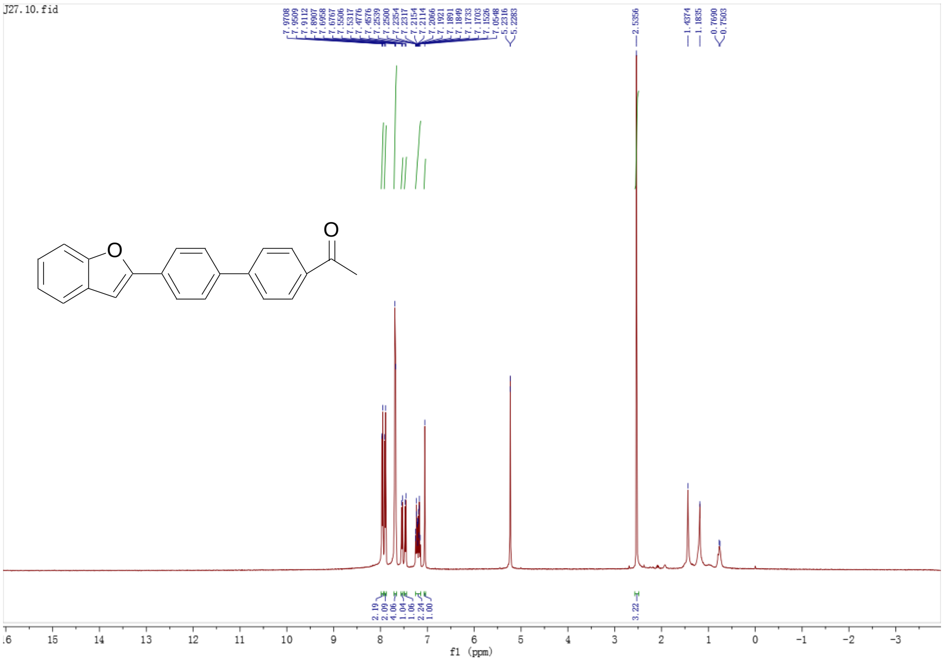

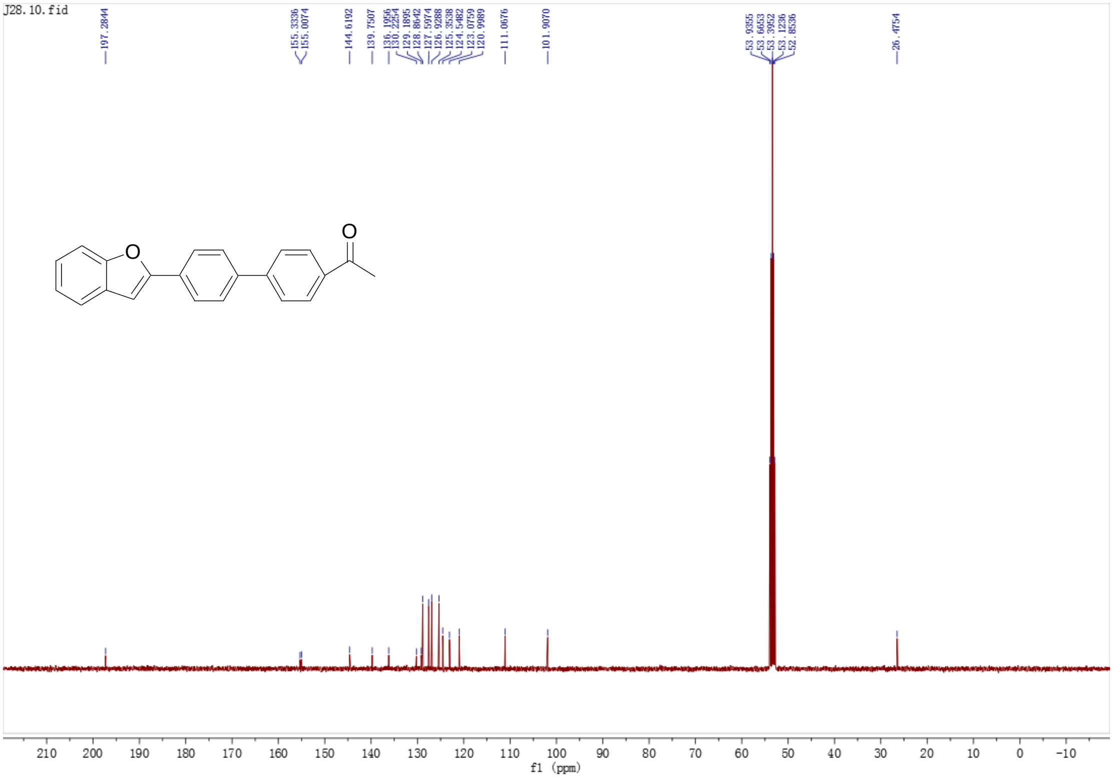
J28.10.fid
197.2844
155.3336
155.0074
144.6192
139.7507
136.1956
130.2254
129.1895
128.8642
127.5974
126.9288
125.3538
124.5482
123.0759
120.9989
111.0676
101.9070
53.9355
53.6653
53.3952
53.1236
52.8536
26.4754
O
f1 (ppm)

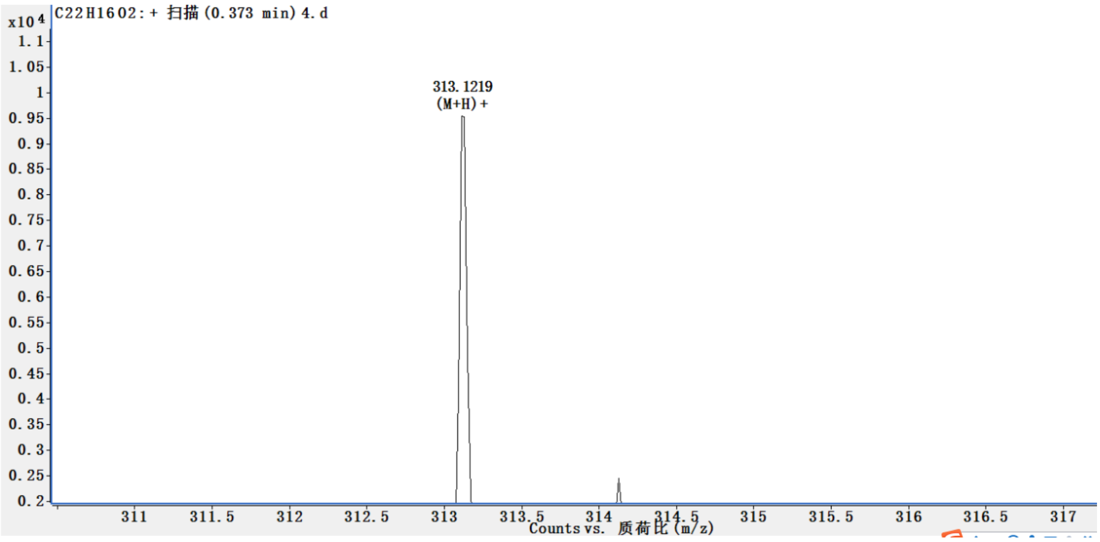
x10 4
C22H16O2: + 扫描 (0.373 min) 4.d
313.1219
(M+H)+
Counts vs. 质荷比 (m/z)

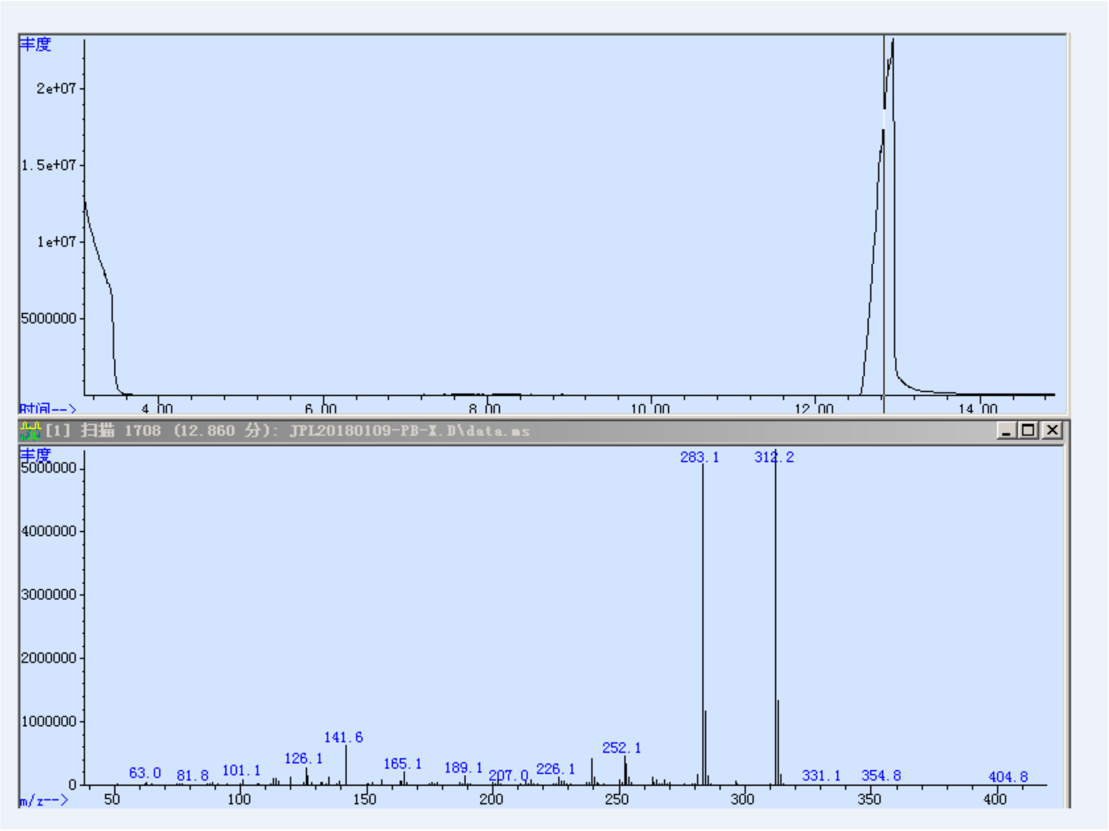

3. ^{1}H-NMR, ^{13}C-NMR, HRMS and GC-MS of 2-(4'-Propyl-biphenyl-4-yl)-benzofuran (solvent:chloroform-d)

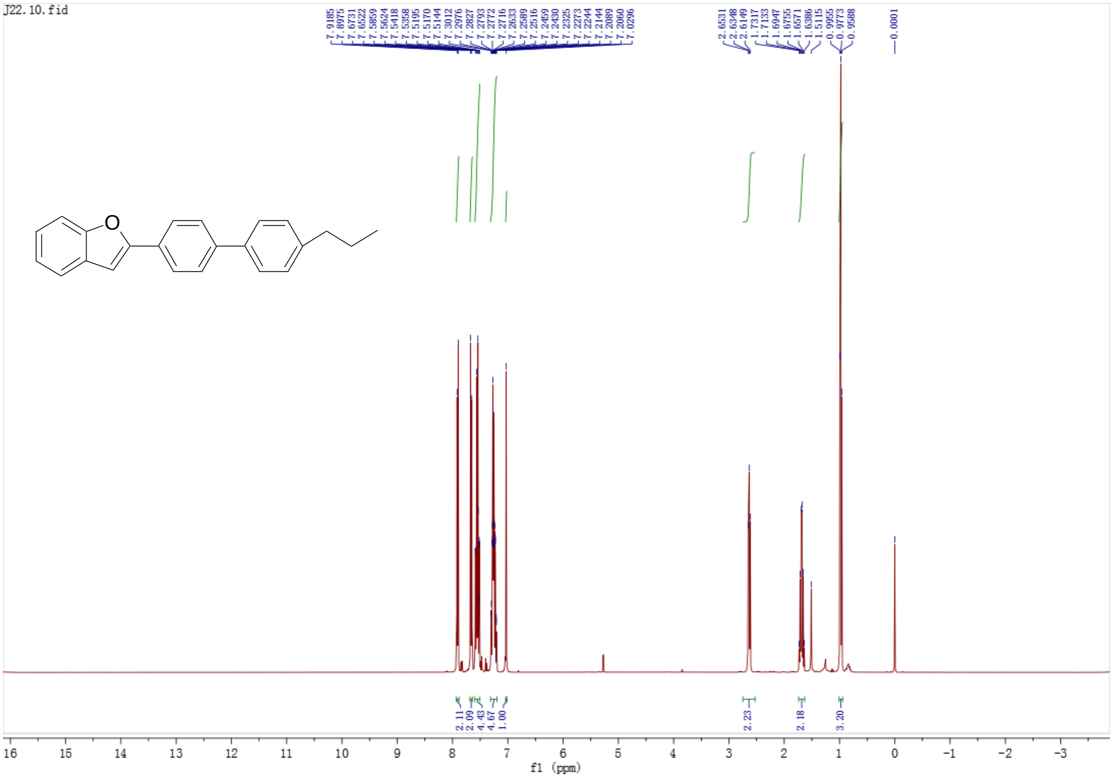

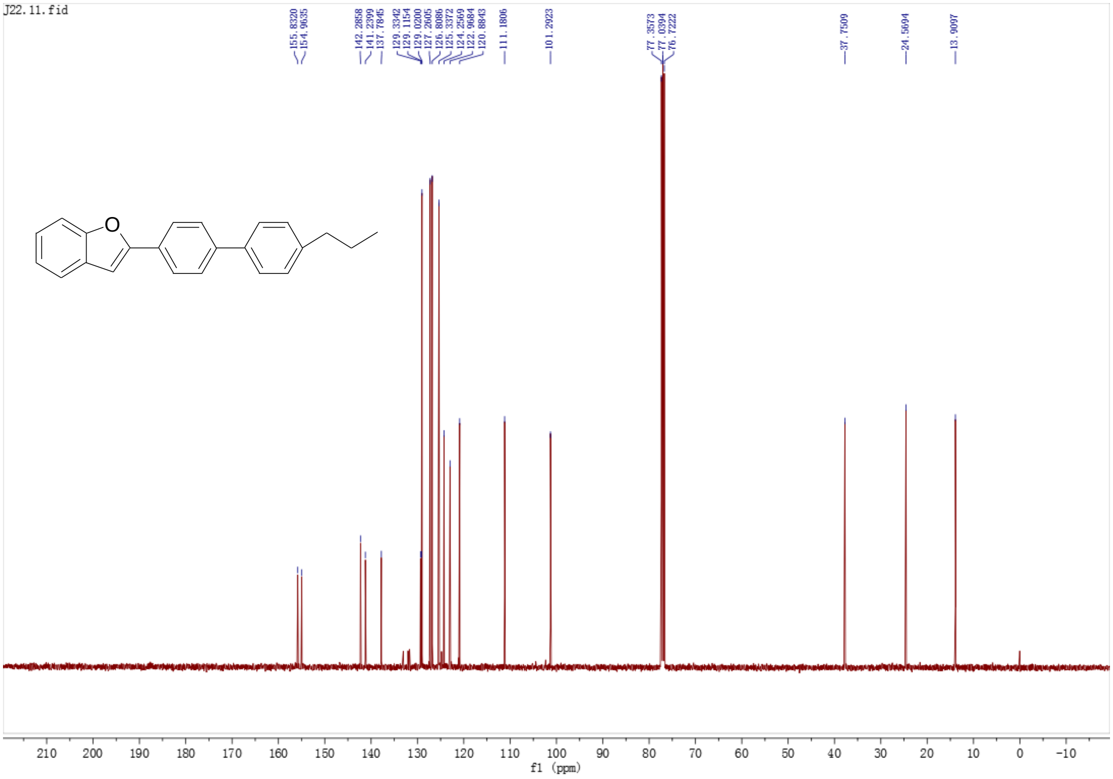
J22.11.fid
155.8320
154.9635
142.2858
141.2399
137.7845
129.3342
129.1154
129.0200
127.2605
126.8086
125.3372
124.2569
122.9684
120.8843
111.1806
101.2923
77.3573
77.0394
76.7222
37.7509
24.5694
13.9097
O
f1 (ppm)

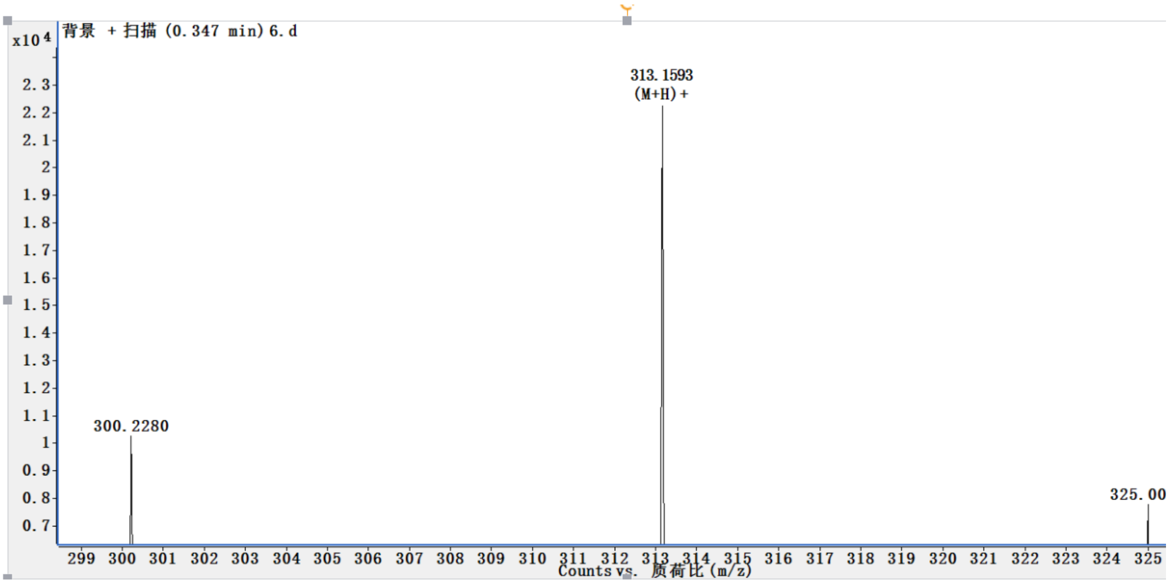
x10 4
背景 + 扫描 (0.347 min) 6.d
313.1593
(M+H)+
300.2280
325.00
Counts vs. 质荷比 (m/z)

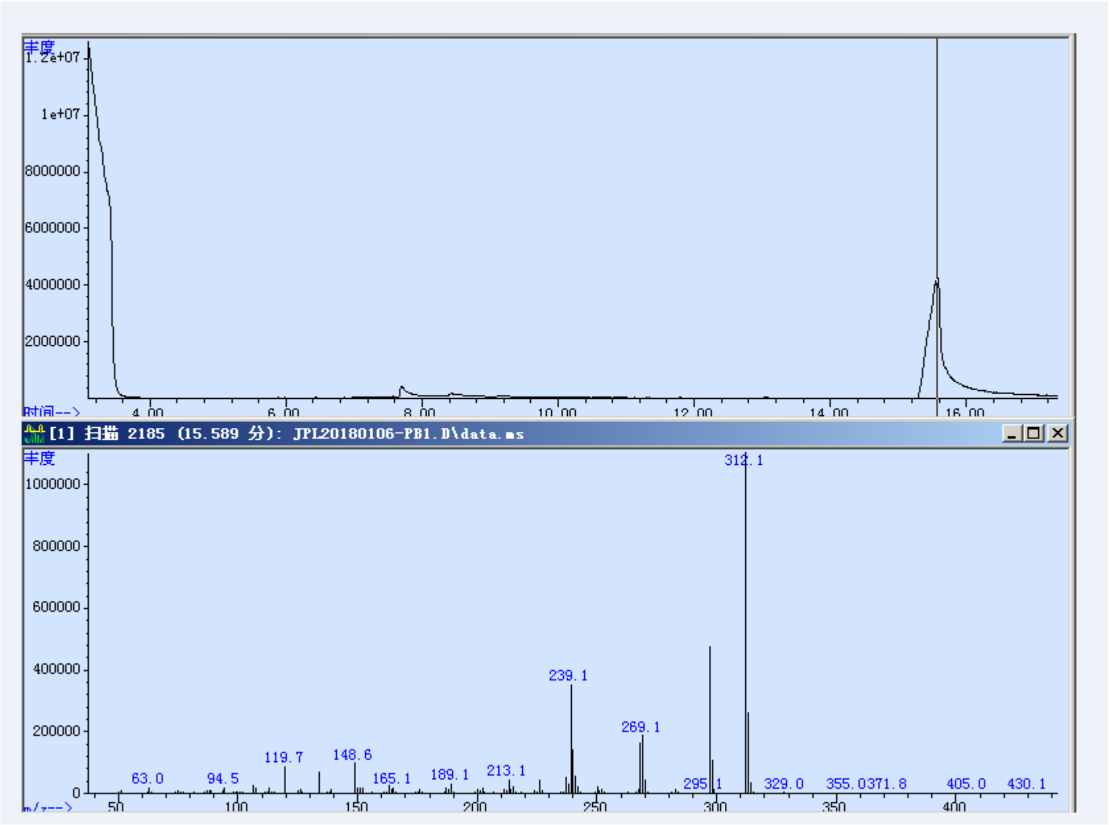

4. ^{1}H-NMR, ^{13}C-NMR, HRMS and GC-MS of 2-(3'-Methyl-biphenyl-4-yl)-benzofuran（solvent:chloroform-d）

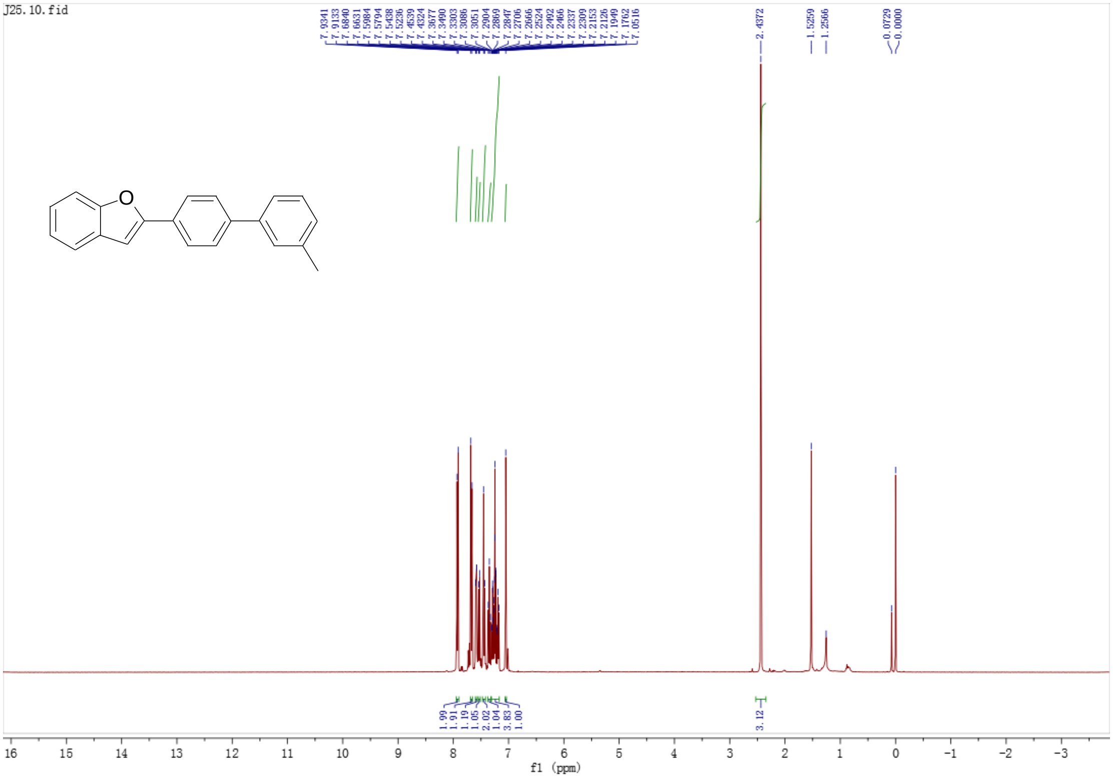

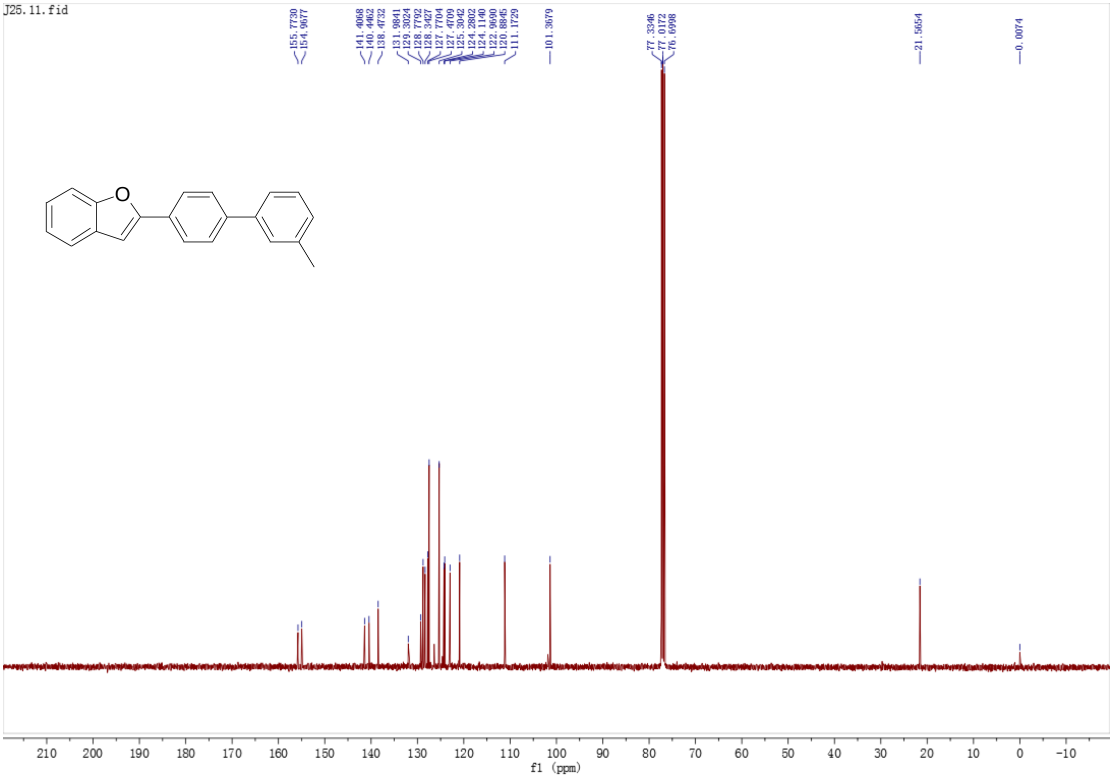

J25.11.fid
155.7730
154.9677
141.4068
140.4462
138.4732
131.9841
129.3024
128.7792
128.3427
127.7704
127.4709
125.3042
124.2802
124.1140
122.9690
120.8845
111.1729
101.3679
77.3346
77.0172
76.6998
21.5654
0.0074
f1 (ppm)

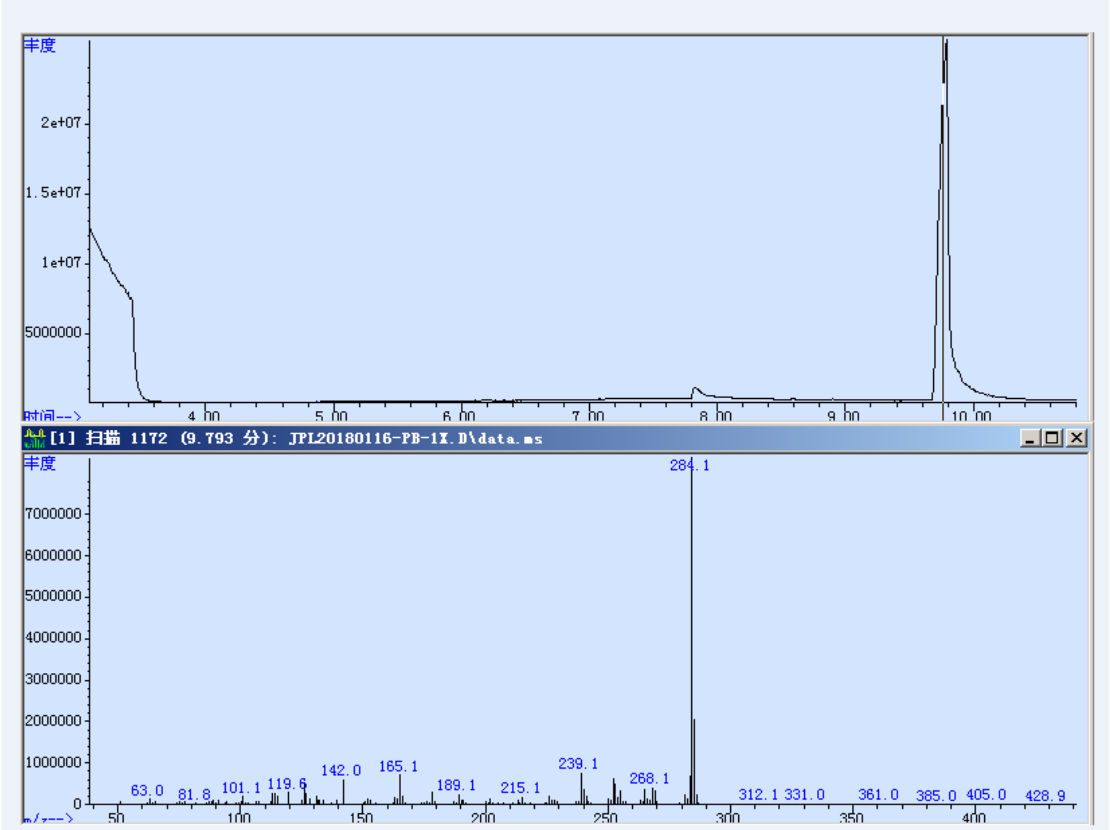

丰度
时间-->
[1] 扫描 1172 (9.793 分): JPL20180116-PB-1X.D\data.ms
284.1
63.0
81.8
101.1
119.6
142.0
165.1
189.1
215.1
239.1
268.1
312.1
331.0
361.0
385.0
405.0
428.9
m/z-->

5. ^{1}H-NMR, ^{13}C-NMR, HRMS, GC-MS of 2-(2'-Methyl-biphenyl-4-yl)-benzofuran (solvent:chloroform-d)

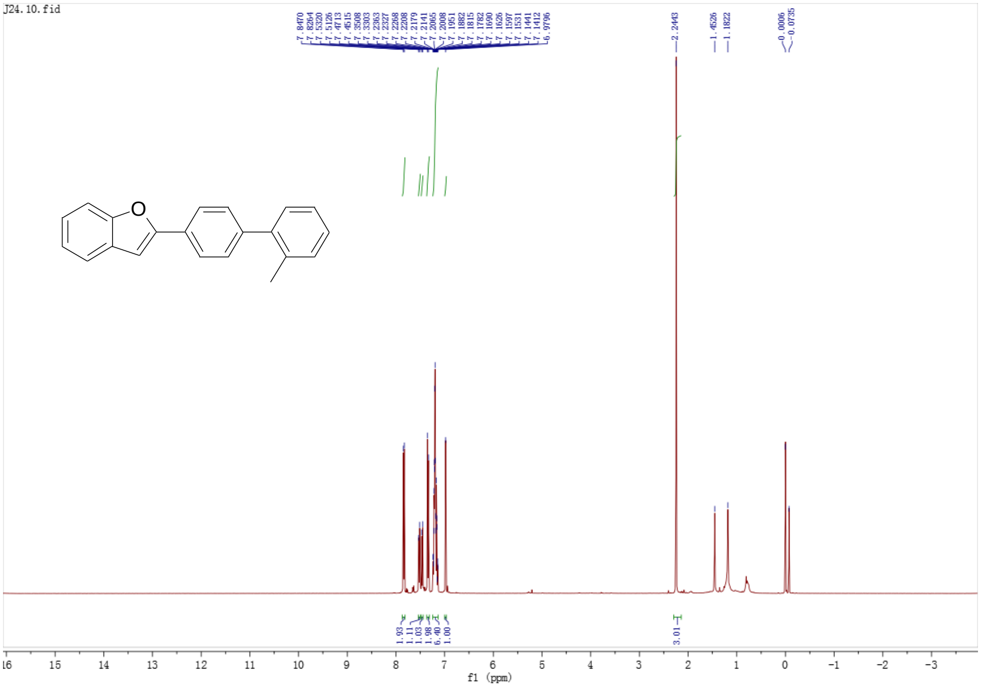

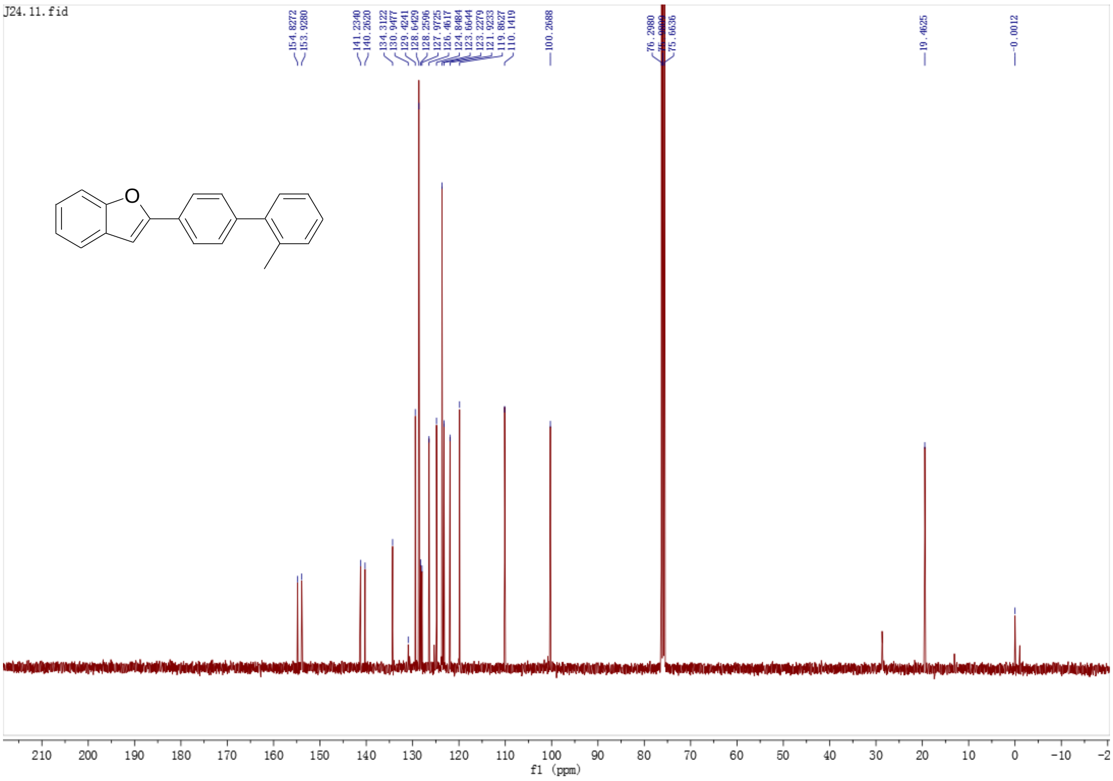

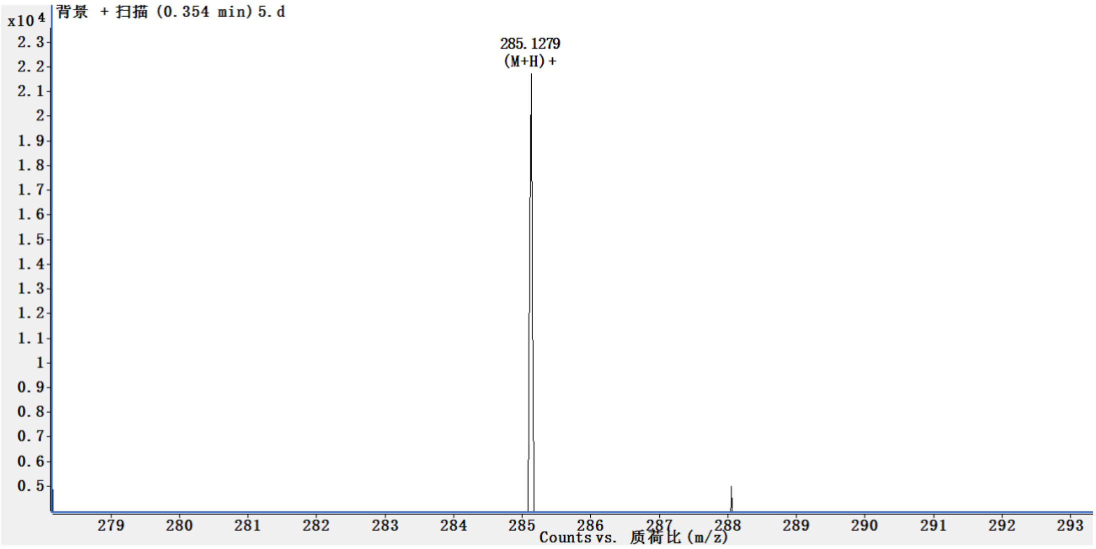

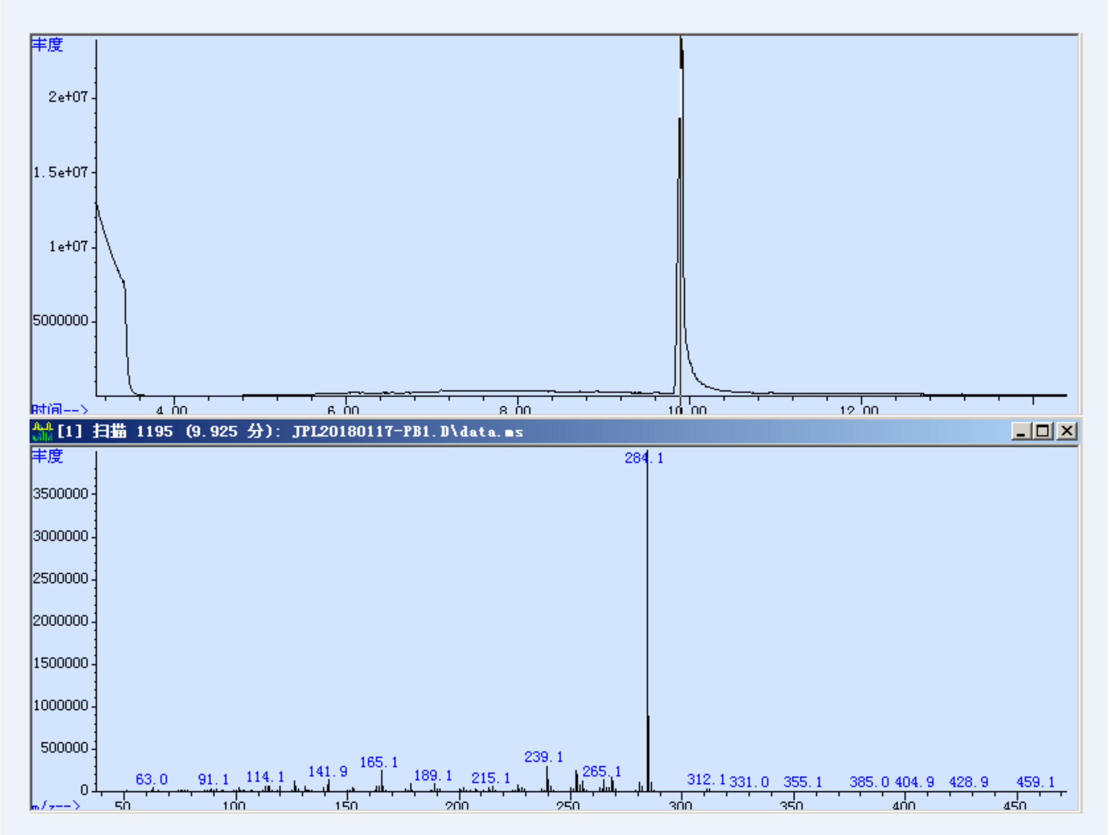

6. ^{1}H-NMR, ^{13}C-NMR, HRMS, GC-MS of
2-(3',4'-Difluoro-biphenyl-4-yl)-benzofuran (solvent:chloroform-d)

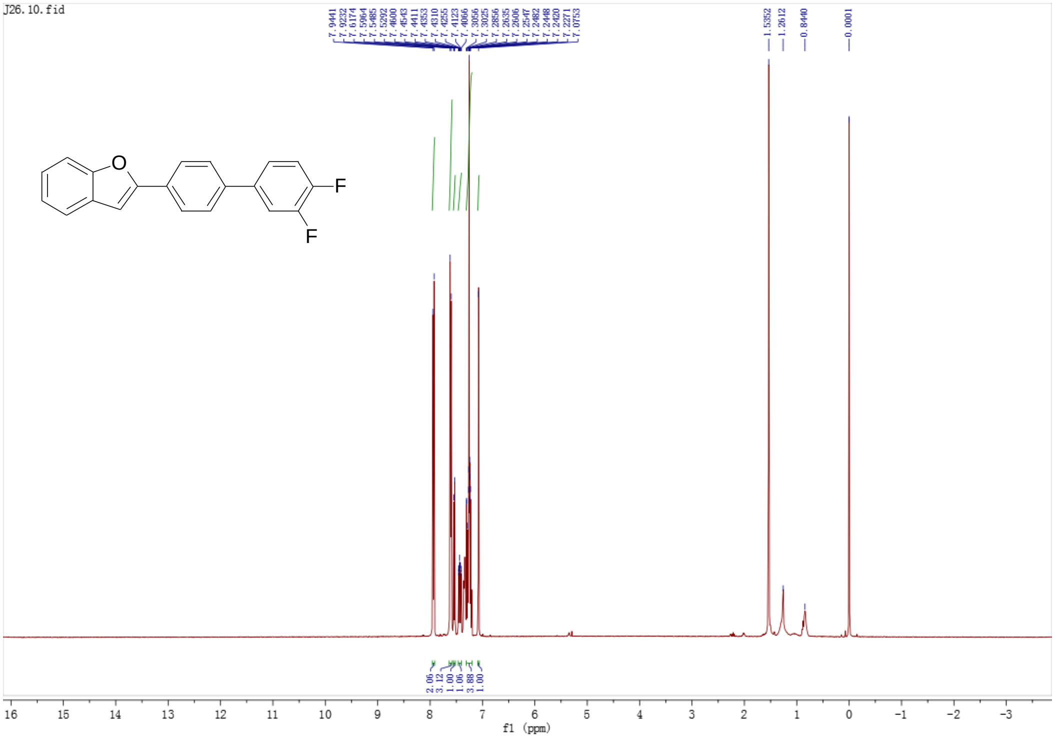
J26.10.fid
7.9441
7.9232
7.6174
7.5964
7.5485
7.5292
7.4600
7.4543
7.4411
7.4353
7.4310
7.4255
7.4123
7.4066
7.3056
7.3025
7.2856
7.2635
7.2606
7.2547
7.2482
7.2448
7.2420
7.2271
7.0753
1.5352
1.2612
0.8440
0.0001
F
F
O
2.06
3.12
1.00
1.06
3.88
1.00
16 15 14 13 12 11 10 9 8 7 6 5 4 3 2 1 0 -1 -2 -3
f1 (ppm)

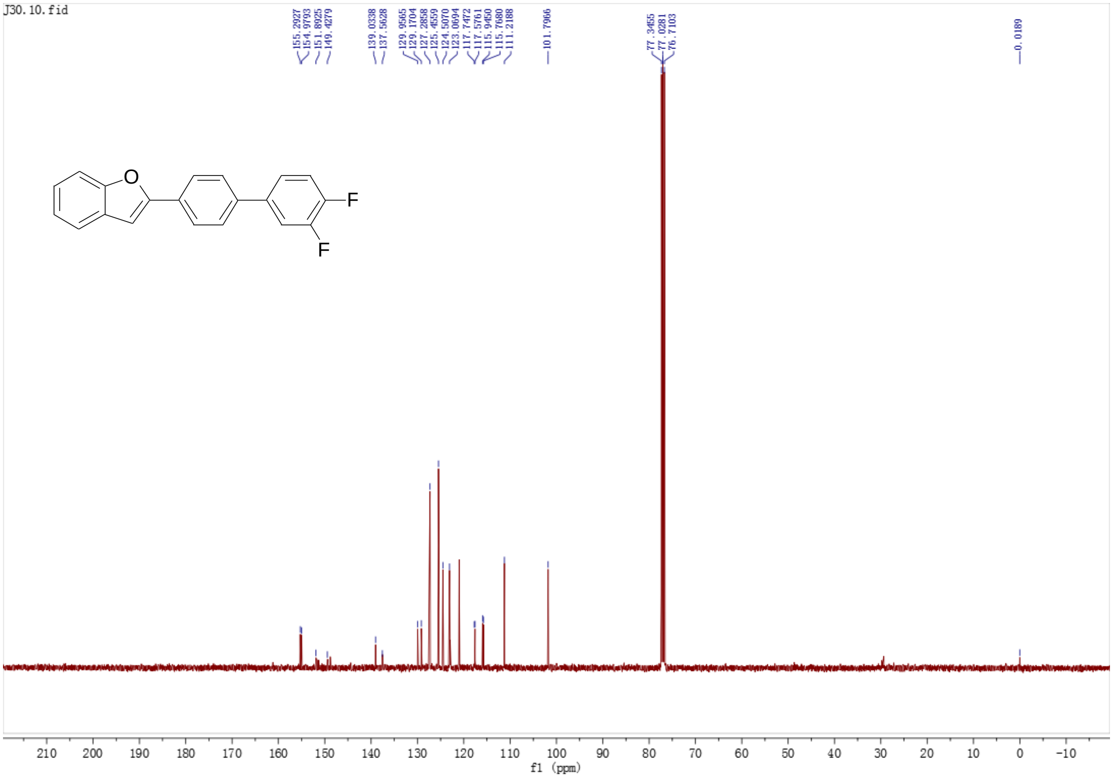
J30.10.fid
155.2927
154.9793
151.8925
149.4279
139.0338
137.5628
129.9565
129.1704
127.2858
125.4559
124.5070
123.0694
117.7472
117.5761
115.9450
115.7680
111.2188
101.7966
77.3455
77.0281
76.7103
0.0189
O
F
F
210 200 190 180 170 160 150 140 130 120 110 100 90 80 70 60 50 40 30 20 10 0 -10
f1 (ppm)

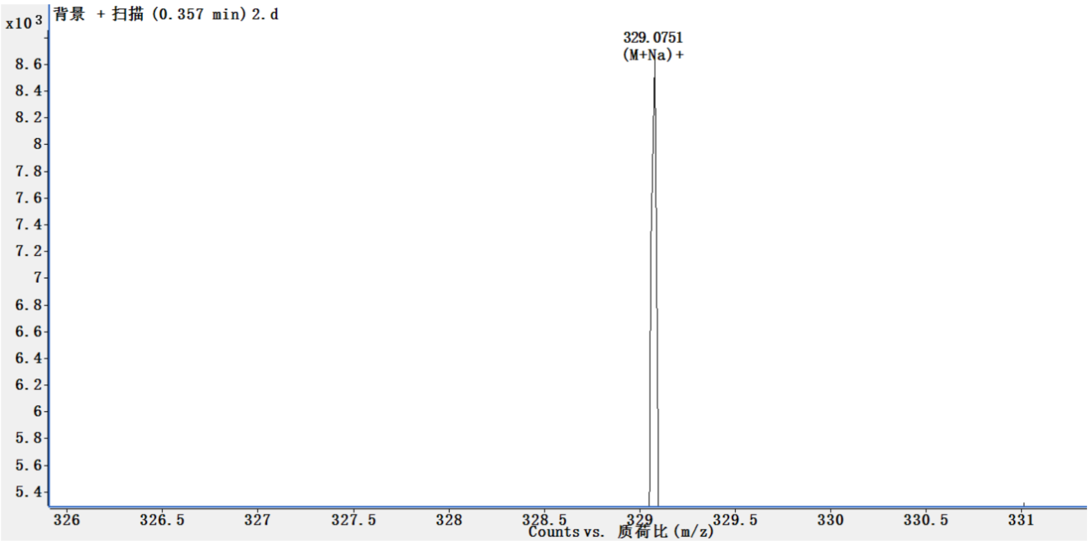

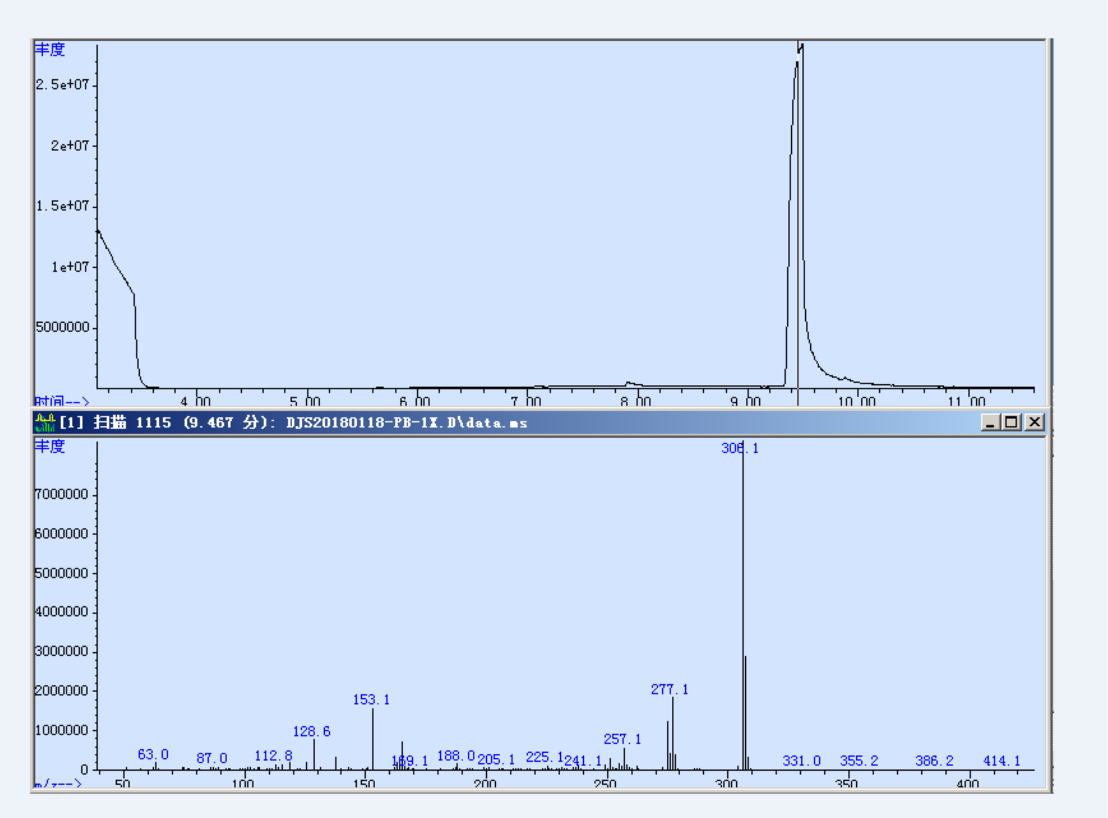

7. ^{1}H-NMR, ^{13}C-NMR, HRMS and GC-MS of 2-(3',5'-Difluoro-biphenyl-4-yl)-benzofuran (solvent:chloroform-d)

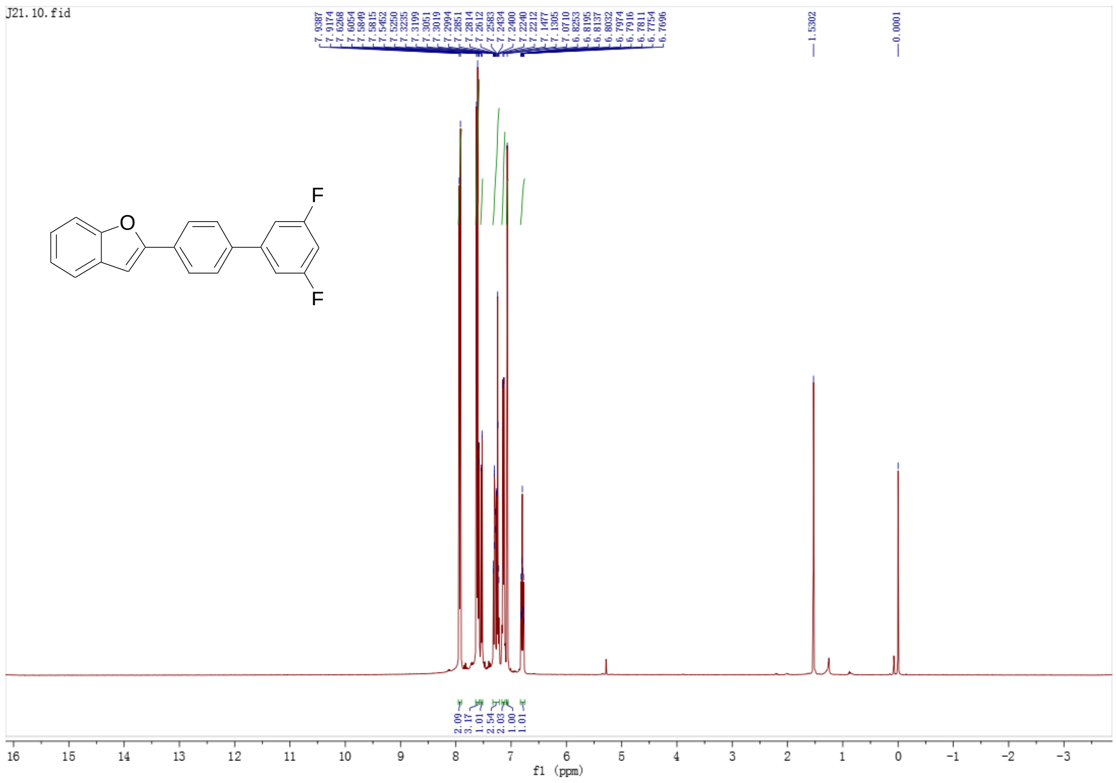

J21.10.fid
f1 (ppm)

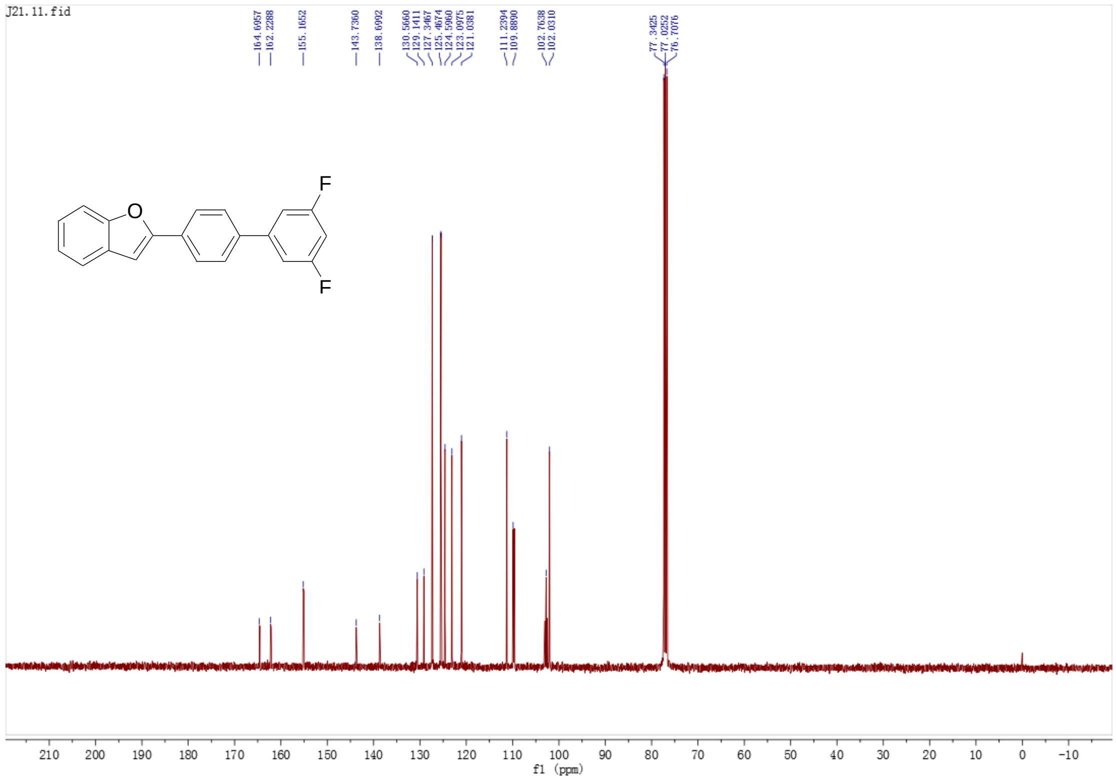

J21.11.fid
f1 (ppm)

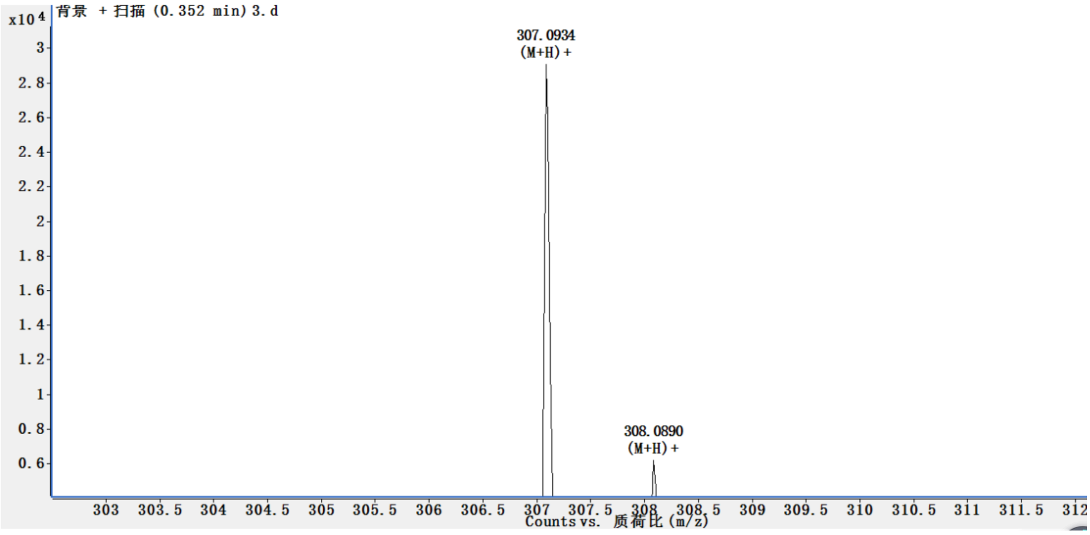
x10 4
背景 + 扫描 (0.352 min) 3.d
307.0934
(M+H)+
308.0890
(M+H)+
3
2.8
2.6
2.4
2.2
2
1.8
1.6
1.4
1.2
1
0.8
0.6
303 303.5 304 304.5 305 305.5 306 306.5 307 307.5 308 308.5 309 309.5 310 310.5 311 311.5 312
Counts vs. 质荷比 (m/z)

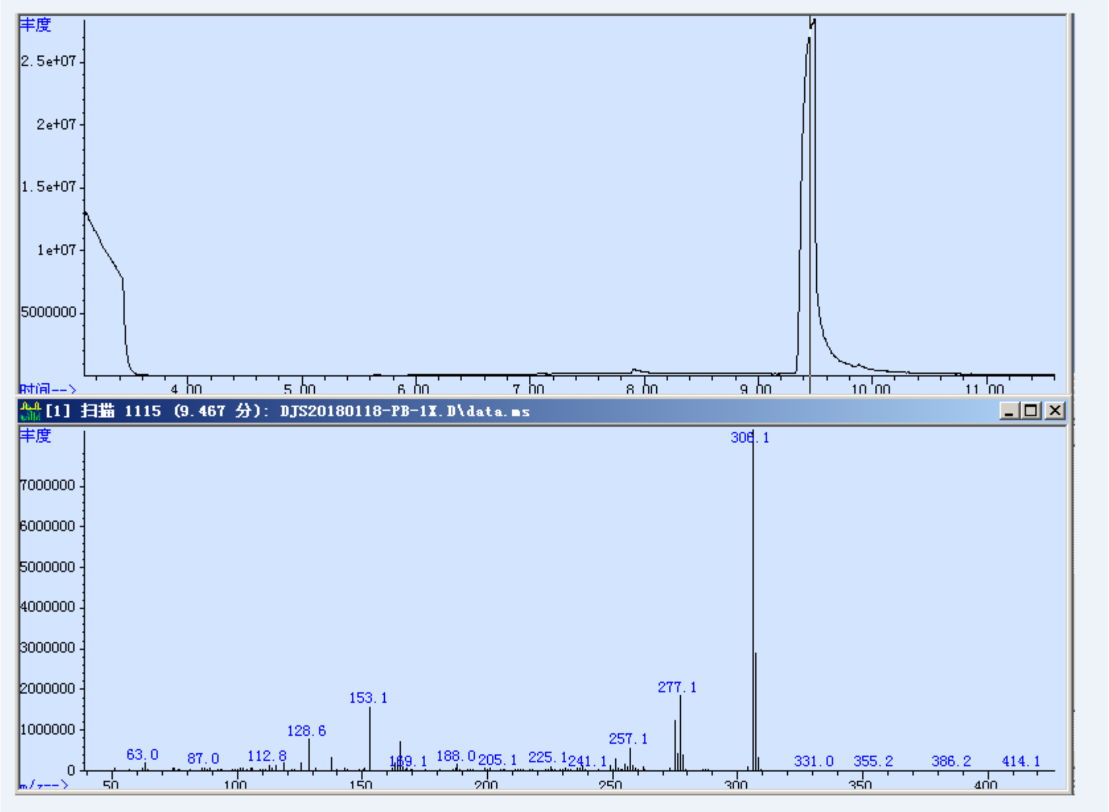
丰度
2.5e+07
2e+07
1.5e+07
1e+07
5000000
时间-->
4.00 5.00 6.00 7.00 8.00 9.00 10.00 11.00
[1] 扫描 1115 (9.467 分): DJS20180118-PB-1X.D\data.ms
丰度
7000000
6000000
5000000
4000000
3000000
2000000
1000000
0
306.1
277.1
153.1
128.6
257.1
63.0
87.0
112.8
188.0
205.1
225.1
241.1
331.0
355.2
386.2
414.1
m/z-->
50 100 150 200 250 300 350 400
